# A role for PAX8 in the tumorigenic phenotype of ovarian cancer cells

**DOI:** 10.1186/1471-2407-14-292

**Published:** 2014-04-26

**Authors:** Tina Di Palma, Valeria Lucci, Tiziana de Cristofaro, Maria Grazia Filippone, Mariastella Zannini

**Affiliations:** 1 IEOS, Institute of Experimental Endocrinology and Oncology ‘G. Salvatore’, National Research Council, Naples, Italy; 2 Department of Molecular Medicine and Medical Biotechnology, University of Naples Federico II, Naples, Italy

**Keywords:** PAX8, Ovarian cancer, shRNA, Gene silencing

## Abstract

**Background:**

PAX8 is a member of the paired box (Pax) multigene family of transcription factors, which are involved in the developmental and tissue-specific control of the expression of several genes in both vertebrates and invertebrates. Previously, several studies reported that PAX8 is expressed at high levels in specific types of tumors. In particular, PAX8 has been recently reported to be conspicuously expressed in human ovarian cancer, but the functional role of PAX8 in the carcinogenesis of this type of tumor has not been addressed. In this study, we investigated the contribution of PAX8 in ovarian cancer progression.

**Methods:**

Stable PAX8 depleted ovarian cancer cells were generated using short hairpin RNA (shRNA) constructs. PAX8 mRNA and protein were detected by RT-PCR, immunoblot and immunofluorescence. Cell proliferation, motility and invasion potential of PAX8 silenced cells were analyzed by means of growth curves, wound healing and Matrigel assays. In addition, PAX8 knockdown and control cells were injected into nude mice for xenograft tumorigenicity assays. Finally, qPCR was used to detect the expression levels of EMT markers in PAX8-overexpressing and control cells.

**Results:**

Here, we show that PAX8 plays a critical role in the migration, invasion and tumorigenic ability of ovarian cancer cells. Our results show that RNA interference-mediated knockdown of PAX8 expression in SKOV-3 ovarian cancer cells produces a significant reduction of cell proliferation, migration ability and invasion activity compared with control parental SKOV-3 cells. Moreover, PAX8 silencing strongly suppresses anchorage-independent growth *in vitro*. Notably, tumorigenesis *in vivo* in a nude mouse xenograft model is also significantly inhibited.

**Conclusions:**

Overall, our results indicate that PAX8 plays an important role in the tumorigenic phenotype of ovarian cancer cells and identifies PAX8 as a potential new target for the treatment of ovarian cancer.

## Background

Ovarian cancer accounts for approximately 3% of all cancers in women and has the highest mortality of all cancers of the female reproductive system. This reflects, in part, a lack of early symptoms and proven ovarian cancer screening tests. Malignant surface epithelial tumors (carcinomas) are the most common ovarian cancers, accounting for 90% of cases. These tumors differentiate during malignant transformation into four major histotypes: serous, mucinous, endometrioid and clear cell. The overall picture suggests that ovarian cancer, like other cancers, is a spectrum of diseases and not a single disease entity [1]. Recently, gene expression profiling studies have indicated that the transcription factor PAX8 is a potential diagnostic marker for ovarian carcinoma [2].

PAX8 is a member of the PAX gene family, consisting of nine well-described transcription factors (PAX1-9). The temporal and spatial expression patterns of PAX genes are tightly regulated, and their expression is observed primarily during fetal development [3]. In most cases, PAX gene expression attenuates when development is complete, but in a few tissues, it persists into adult life. However, abnormal cell growth and proliferation is often associated with high expression levels of PAX genes [4]. Nevertheless, the precise role that PAX genes play in cancer is still unclear. Cancer-promoting PAX genes might not themselves be responsible for cells shifting to a tumorigenic state, but following tumor onset, PAX genes clearly play important roles in the acquisition of characteristics that define malignancy. In fact, overexpression of PAX proteins *per se* does not appear to be an initiating or transforming molecular event in tumor pathogenesis, but it facilitates malignant development through the effects of PAX genes on apoptosis resistance, tumor cell proliferation and migration, and repression of terminal differentiation [4].

PAX8 plays a key role in thyrocyte differentiation [5]. It is expressed during the organogenesis of the thyroid gland, Mullerian tract, and kidney, as well as in the adult thyroid and kidney [6]. Knockout mice lacking PAX8 have a smaller thyroid, with normal calcitonin-producing parafollicular C cells but no follicular cells; thus, they suffer from severe hypothyroidism [7]. Congenital hypothyroidism is caused by several genetic defects, and among these there are mutations of the PAX8 gene [8]. In addition to hypothyroidism, PAX8 plays a role in the progression of follicular thyroid carcinomas and adenomas [9] and is overexpressed in the majority of gliomas, Wilms tumors and well-differentiated pancreatic neuroendocrine tumors [10-12]. Interestingly, aberrant expression of PAX8 has been reported in epithelial ovarian cancer [13], and it was described as one of the top 40 genes specifically upregulated in different types of ovarian carcinomas [14]. PAX8 is not expressed in the surface epithelial cells of the ovary; however, recently its expression was found in 96% of serous ovarian carcinomas, in 89% of endometrioid and 100% of clear cell carcinomas, whilst was not detected in mucinous carcinomas [9]. Recently, it has been demonstrated that high-grade serous carcinoma (HGSC) originates in fallopian tubal secretory epithelial cells, which are positive for PAX8 expression [15].

Our studies provide strong evidence that PAX8 plays an important role in the tumorigenicity of ovarian cancer cells both *in vitro* and *in vivo* and identify PAX8 as a major biomarker and target for ovarian cancer.

## Methods

### Cell culture and DNA transfection

The human ovarian carcinoma cell lines SKOV-3, TOV-21G, OVCAR-3, TOV-112D and A2780 were obtained from the CEINGE Cell Culture Facility (Naples, Italy) and were grown in RPMI medium (Euroclone) containing 10% fetal bovine serum (Euroclone). The medullary and cortical cells were kindly provided by Prof. Lucio Nitsch (University of Naples, Italy) and were maintained in CHANG MEDIUM C lyophilized kit (Irvine Scientific). The nontumorigenic ovarian cells IOSE-80PC were obtained by Canadian Ovarian Tissue Bank and were grown in medium 199:MCDB 105 (Sigma-Aldrich) containing 10% fetal bovine serum. For stable transfection experiments, cells were plated at 5 × 10^5^ cells/100-mm tissue culture dish 24 h prior to transfection. Transfections were carried out with the Lipofectamine (Invitrogen) and FUGENE reagent (Promega) for SKOV-3 and IOSE-80PC cells, respectively, according to the manufacturer's directions. Forty-eight hours later, transfected cells SKOV-3 and IOSE-80PC were selected in the presence of 0.4 μg/ml of puromycin (Sigma-Aldrich) and 0.2 mg/ml of G418 (Gibco), respectively.

### RNA extraction, RT-PCR analysis and quantitative real time RT–PCR

Total RNA was extracted using TRIzol reagent (Invitrogen) and cDNA was synthesized using iScript cDNA Synthesis kit according to the manufacturer’s instructions (Biorad). Subsequently, cDNA was used for each PCR reaction with each primer pair. The PAX8 specific primers designed to detect PAX8 splice variants were previously described [13]. Real time RT–PCR analysis was performed using IQ™ SYBR Green PCR Master Mix (Biorad) in a iCycler IQ™ real-time detection system (Biorad).

### Cell extracts and Western blot

Cell extracts and western blot were carried out as previously described [16].

### shRNA, plasmid and antibodies

Five shRNA targeting PAX8, Mission shRNA lentiviral plasmids (SHCLNG-NM_003466, Sigma-Aldrich) and MISSION Non-targeting shRNA control vector (SHC002, Sigma-Aldrich) were used. The 3XFLAG-Pax8 expression vector was previously described [16]. The antibodies used for immunofluorescence and immunoblotting were: Pax8 (kindly provided by Prof. R. Di Lauro), fibronectin clone IST4 (Sigma-Aldrich), vimentin, twist, vinculin, actin and α-tubulin (Santa Cruz Biotechnology).

### Cell proliferation and invasion assay

To evaluate cell growth, SKOV-3, SKOVCtrl-, siCl32, and siCl48 cells were plated at 8 × 10^4^ cells per 60-mm plate. The medium was changed every 24 h, and every 24 h cells were collected and counted. Cell invasion assay was examined using a reconstituted extracellular matrix (Matrigel; BD Biosciences). Filters (8 μm pore size) on the bottoms of the upper compartment of the transwells (6,5 mm; Corning) were coated with 2 mg/ml of matrigel. 2 × 10^5^ cells were suspended in 100 μl of RPMI with 0.2% FBS. The cells were then plated onto the coated wells and incubated at 37°C for 16 h. Medium in the lower compartment was supplemented with 5% FBS as a chemoattractant. Noninvading cells were removed from the top of the wells with a moistened cotton swab. Cells that penetrated the membrane were fixed with 11% glutaraldehyde and stained with 0.1% crystal violet. The concentration of solubilized crystal violet in 10% acetic acid was evaluated as absorbance at 590 nm. Results are ± SD of three independent experiments.

### Immunofluorescence and Confocal Laser Scanning Microscopy

Cells were grown directly on glass coverslips for 48-72 h, fixed in 4% paraformaldehyde in PBS for 20 min at room temperature, permeabilized for 10 min in 0.2% Triton X-100 in PBS, and incubated for 60 min in 0.5% BSA (bovine serum albumin) in PBS. The coverslips were subsequently incubated at 4°C for 1 h with rabbit polyclonal anti-PAX8 diluted 1:1000 in 0.5% BSA in PBS and, after PBS washing, incubated for 30 min with Alexa Fluor-594 goat anti-rabbit IgG (Vinci Biochem) diluted 1:200 in 0.5% BSA in PBS. After final washings with PBS, the coverslips were mounted on a microscope slide using a 50% solution of glycerol in PBS. Images were collected with a Zeiss LSM 510 confocal laser scanning microscope, equipped with a 543 nm HeNe laser, and a Plan-Apochromat 63/1.4 oil immersion objective. Emitted fluorescence was detected using LP 560 long pass filter for TRITC.

### Wound-healing assay

Confluent SKOVCtrl-, siCl32, and siCl48 cells plated on tissue culture dishes were wounded by manual scratching with 200-μl pipette tip, washed with PBS and incubated at 37°C in complete media. At the indicated time points, phase contrast images at specific wound sites were captured.

### Anchorage-independent growth in soft agar

Cells were mixed in RPMI 2X (Sigma-Aldrich Aldrich), tryptose phosphate buffer, and 1.25% of Noble Agar (Difco Laboratories Inc.) and plated in 60-mm dishes on the top of 1% agar base. The colonies were allowed to grow in incubator at 37°C, 5% CO2 for 2 to 3 weeks. The images of cell colonies were captured with an inverted microscope.

### Animal experiments

All animal studies were conducted at Biogem Scarl Ariano Irpino, AV (Italy), Preclinical Research and Development Service. Nude female NOD-SCID mice (NOD-CB17/PRKDC/J) were purchased from Charles River Laboratories International, Wilmington, MA. Animals have been housed and used following the rules of the Italian laws (DL.vo N° 116 **-** 27/01/1992 and related) and of the EU directive (2010/63/UE **-** 22/09/2010) on the protection of animals used for experimental purposes. All the in vivo procedures were in compliance with the *Guide for the Care and Use of Laboratory Animals* (United States National Research Council, 1996). All the *in vivo* experimental activities were evaluated and approved by the Committee for the Ethics of the Experimentations on Animals (CESA) of Biogem (ID code 4215) and were authorized by the Italian Minister of Health.

To generate xenografts, human ovarian cancer cells were cultured in DMEM with 10% heat-inactivated FBS. 24 six-week-old nude female NOD-SCID mice (NOD-CB17/PRKDC/J) (Charles River Laboratories International) were randomly assigned to four groups: the SKOV3 group (n = 6), SKOV3Ctrl- group (n = 6), siCl32 group (n = 6) and siCl48 group (n = 6). They were injected subcutaneously in the both flanks with 7×10^6^ cells suspended in 0.2 ml PBS/Matrigel Matrix GF (1:1) (BD Biosciences). Mice were daily monitored for clinical signs and mortality. Body weight recordings were carried out weekly. Tumor growth was measured twice a week with a Mitutoyo caliper. The formula TV (mm^3^) = [length (mm) × width (mm)^2^]/2 was used. At the end of the study mice were sacrificed by cervical dislocation.

## Results

### PAX8 expression in human ovarian cancer cell lines

**Figure 1:**
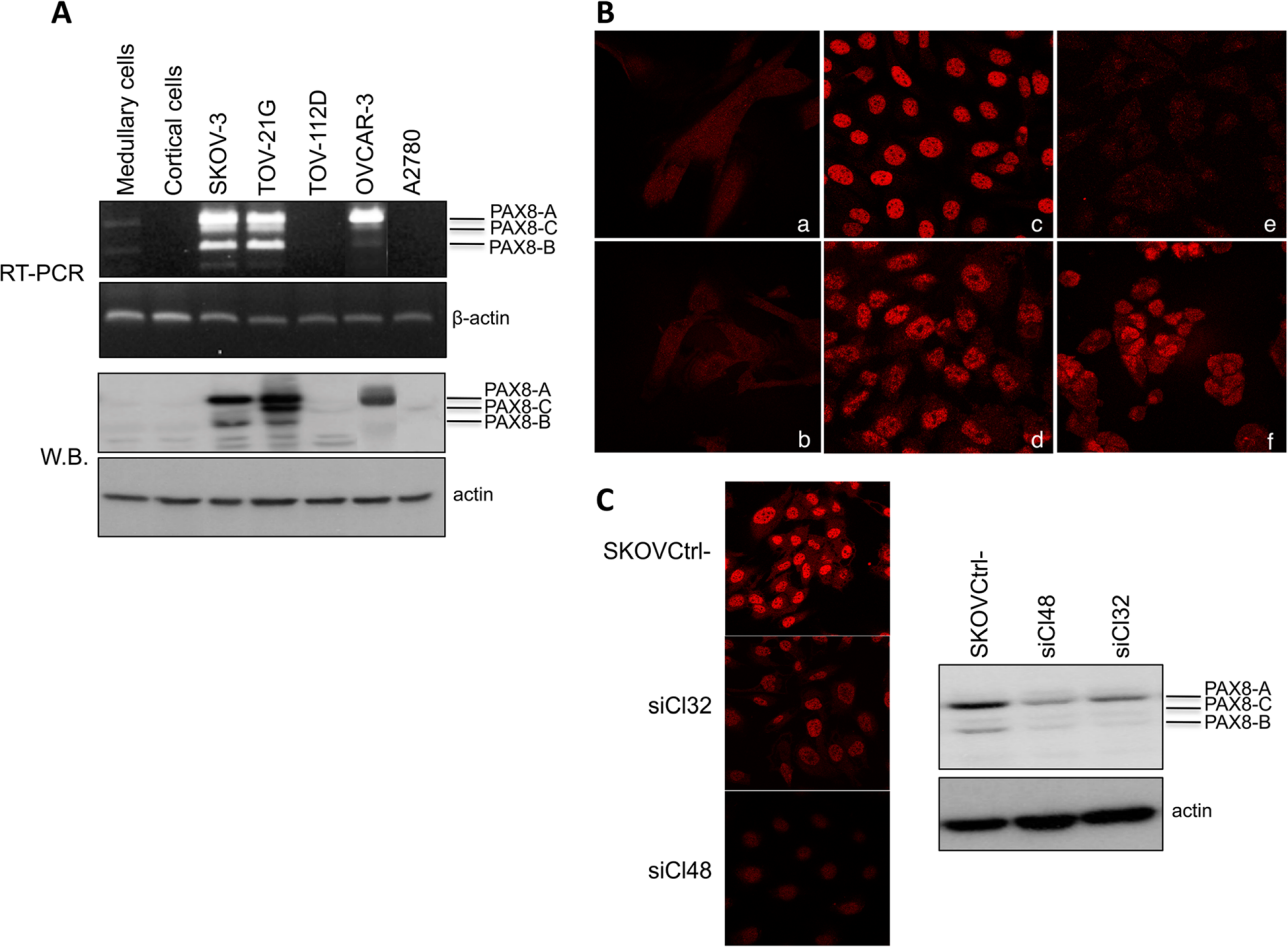
**Expression of PAX8 in ovarian cancer cell lines. (A)** RT-PCR was performed using total RNA from the indicated cell lines. β-actin mRNA was amplified as control. Total protein extracts prepared from the same cell lines were separated on SDS-PAGE and subjected to Western blot analysis with a specific anti-PAX8 antibody. The hybridization with actin assessed the protein uniform loading and integrity. PAX8 splice variants are indicated in both experiments. **(B, C)** ovarian cells were grown directly on glass coverslips and stained for indirect immunofluorescence with the anti-PAX8 antibody. The fluorescence signals were acquired at a confocal microscope, by line-wise scanning. In panel B cells are: ***a,*** medullary cells; ***b,*** cortical cells; ***c,*** SKOV-3; ***d,*** TOV-21G; ***e,*** TOV-112D; ***f,*** A2780 cells. In panel C, the extent of Pax8 silencing measured by Western blot is shown.

It has been recently reported that PAX8 is expressed in a subset of epithelial tumors [17] and could be a useful marker for the detection and differential diagnosis of ovarian carcinoma [9]. We first examined the expression of PAX8 in several ovarian cancer cell lines by RT-PCR and immunoblotting (Figure 1A). PAX8 is expressed at high levels in SKOV-3, TOV-21G and OVCAR-3 ovarian cancer cell lines, whereas it is undetectable in two primary normal ovarian cultures (medullary and cortical cells) and in the TOV-112D and A2780 ovarian cancer cell lines. The multiple bands detected by RT-PCR and Western blot correspond to different PAX8 isoforms previously described [18]. Furthermore, the subcellular localization of PAX8 protein was analyzed by immunofluorescence and specific staining was observed exclusively in nuclei, as expected (Figure 1B).

To investigate the role of PAX8 in the tumorigenic properties of ovarian cancer cells, we silenced PAX8 expression in the SKOV-3 cell line using shRNA plasmid vectors. Specifically, we stably transfected the cells with a pool of five plasmid vectors, each containing an shRNA targeting a different region of PAX8 cDNA. This strategy enabled us to achieve >70% inhibition of PAX8. Figure 1C shows, by means of indirect immunofluorescence and Western blot with a specific anti-PAX8 antibody, the inhibition of PAX8 expression in two independent representative clones (siCl32 and siCl48), compared to cells transfected with the control vector containing a scrambled shRNA (SKOVCtrl-).

### shRNA-mediated PAX8 knockdown in SKOV-3 cells leads to reduced cell proliferation and suppresses cell migration and invasion

**Figure 2:**
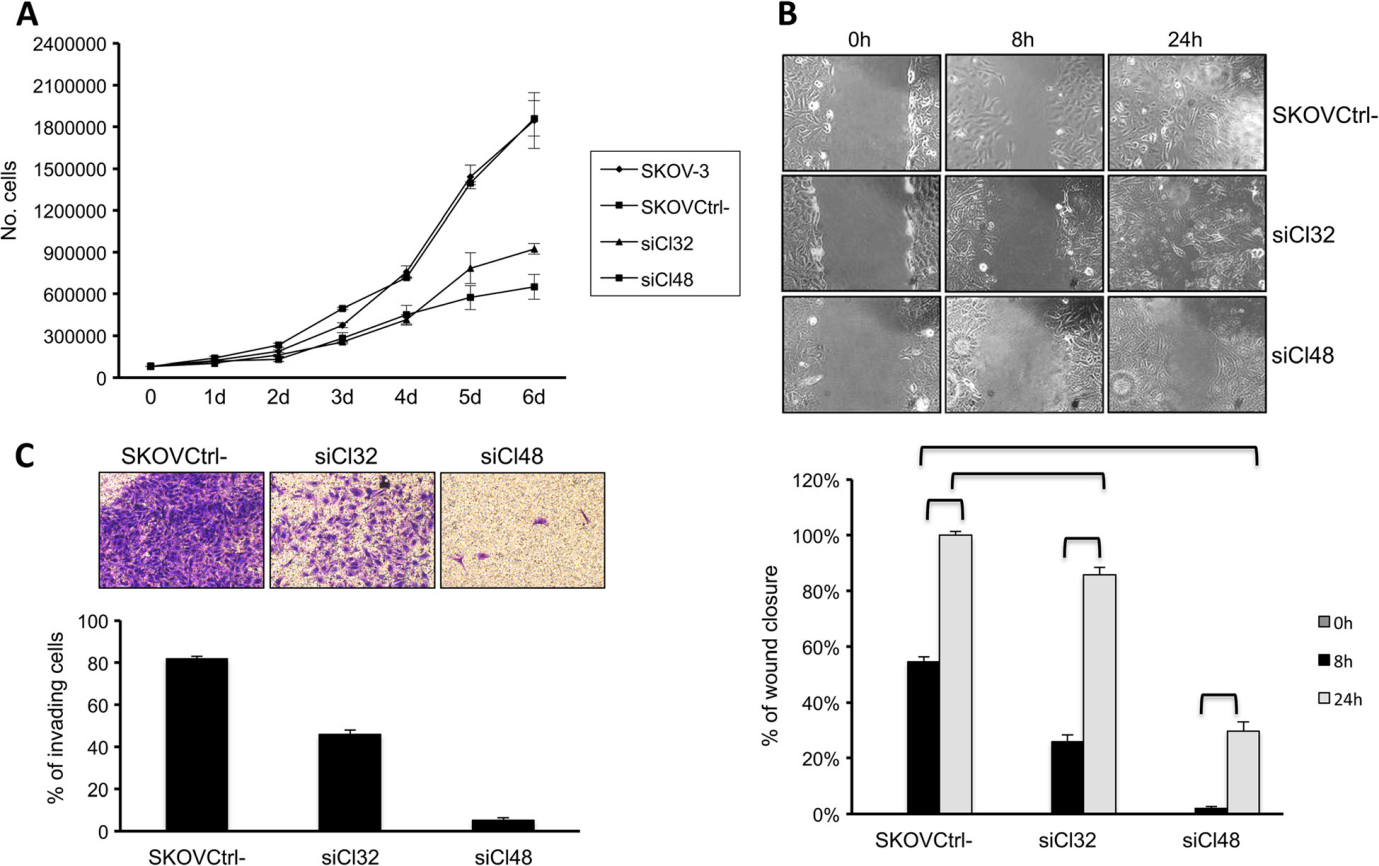
**PAX8 silencing in SKOV-3 cells inhibits cell proliferation, migration, and invasion. (A)** growth curves of SKOV-3, SKOVCtrl-, siCl32, and siCl48 cells are shown. Triplicate of 8 × 10^4^ cells were seeded into 60-mm plate. Cell numbers were counted on days 1, 2, 3, 4, 5 and 6 after seeding. **(B)** wound-healing migration assay for SKOVCtrl-, siCl32, and siCl48 were performed. The healing of the wounds by migrating cells was imaged at time 0, 8 and 24 h (upper panel). Quantitation of the wound-healing migration assay. Wound closure was calculated as the distance covered by cells in relation to the initial wound diameter, as determined at 0 h. Wound closure is expressed as the percentage of the initial wound diameter at 0 h. Data are expressed as the mean ± SD (*Ρ* < 0.01, bottom panel). **(C)** Matrigel invasion assay of SKOVCtrl-, and siPAX8 clones is shown. *Columns,* mean of three independent experiments; *bars,* SD (*Ρ* < 0.05).

To examine whether high levels of PAX8 could directly contribute to the tumorigenicity of ovarian cancer cells, we analyzed whether PAX8 silencing was able to modify the oncogenic properties of SKOV-3 cells. Indeed, growth curve experiments clearly demonstrate that PAX8 expression confers a proliferative advantage to SKOV-3 cells (Figure 2A).

To further study the role of PAX8 in cell migration and invasion, wound healing and transwell assays were performed. In the wound healing assays, we compared the cell mobility of the siCl32 and siCl48 independent stable clones with that of SKOVCtrl- cells. After 8 hours, the area of the wound was significantly re-covered by migrating SKOVCtrl- cells, and after 24 hours the wound area had been completely re-covered (Figure 2B). These cells behaved exactly like SKOV-3 parental cells in all analyses (data not shown). In contrast, the motility of the two siPAX8 clones was significantly decreased and correlated with PAX8 expression levels. Indeed, wound closure of the siCl48 clone (that expresses very low levels of PAX8, see Figure 1C) was not significant after 8 hours and was only partial after 24 hours (Figure 2B), suggesting that PAX8 silencing strongly reduces the migration ability of SKOV-3 ovarian cancer cells. Next, the ability to invade Matrigel was assessed using a Transwell assay (Figure 2C). The invasiveness of PAX8 silenced cells was decreased. In particular, the invasion ability of siCl48 cells was reduced ~8-fold compared to control cells (Figure 2C). All together, these results indicate that PAX8 is involved in cell migration and invasion capabilities of ovarian cancer cells.

### PAX8 is important for anchorage-independent growth and in vivo tumorigenesis

**Figure 3:**
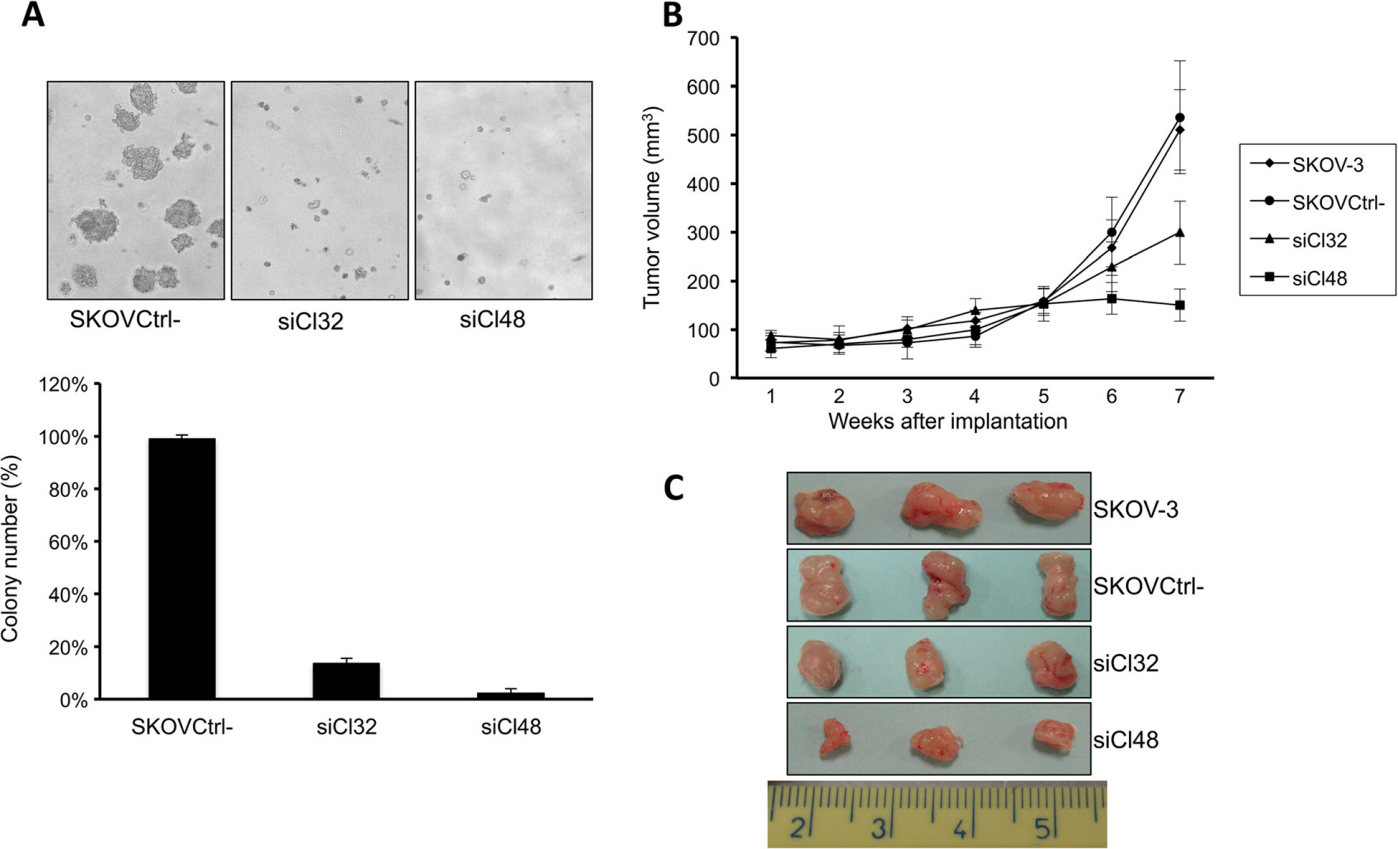
**Loss of PAX8 inhibits anchorage-independent growth in soft agar and suppresses tumorigenesis in nude mice. (A)** soft agar growth of SKOVCtrl-, and siPAX8 cells was assessed and photographed. *Columns,* mean of three independent experiments; *bars,* SD (*Ρ* < 0.05). **(B)** growth curves of tumors in tumor-bearing nude mice injected with SKOV3, SKOVCtrl-, siCl32 and siCl48 cells. Data are plotted as the mean volume ± SD (*n* = 6 for each group). **(C)** representative images of SKOV3, SKOVCtrl-, siCl32 and siCl48 tumors following surgical resection.

To investigate the importance of PAX8 in the tumorigenicity of ovarian cancer cells *in vitro* and *in vivo*, we performed soft agar and nude mice assays. SKOVCtrl- cells grew efficiently in soft agar and formed many colonies, whereas siPAX8 cells exhibited a significant reduction in anchorage-independent growth on soft agar; only small aggregations of cell debris were observed (Figure 3A). These results unambiguously suggest that PAX8 is essential for anchorage-independent growth of SKOV-3 cells. Subsequently, to examine the contribution of PAX8 to *in vivo* tumorigenesis, SKOVCtrl-, siCl32, siCl48 and parental cells were separately injected into the flanks of nude mice and the growth of the tumors was monitored. As shown in Figure 3B, SKOV-3 parental cells formed tumors with the same efficiency as the SKOVCtrl- cells. While siCl32 and siCl48 cells were able to form tumors, the tumors were much smaller, and their size correlated with PAX8 expression levels (Figure 3B). At the end of the experiment, all the tumors were excised and the quantitative analysis is shown in Figure 3C. These data emphasize that PAX8 overexpression is crucial for *in vivo* tumorigenesis.

### PAX8 induces EMT in normal ovarian cells

**Figure 4:**
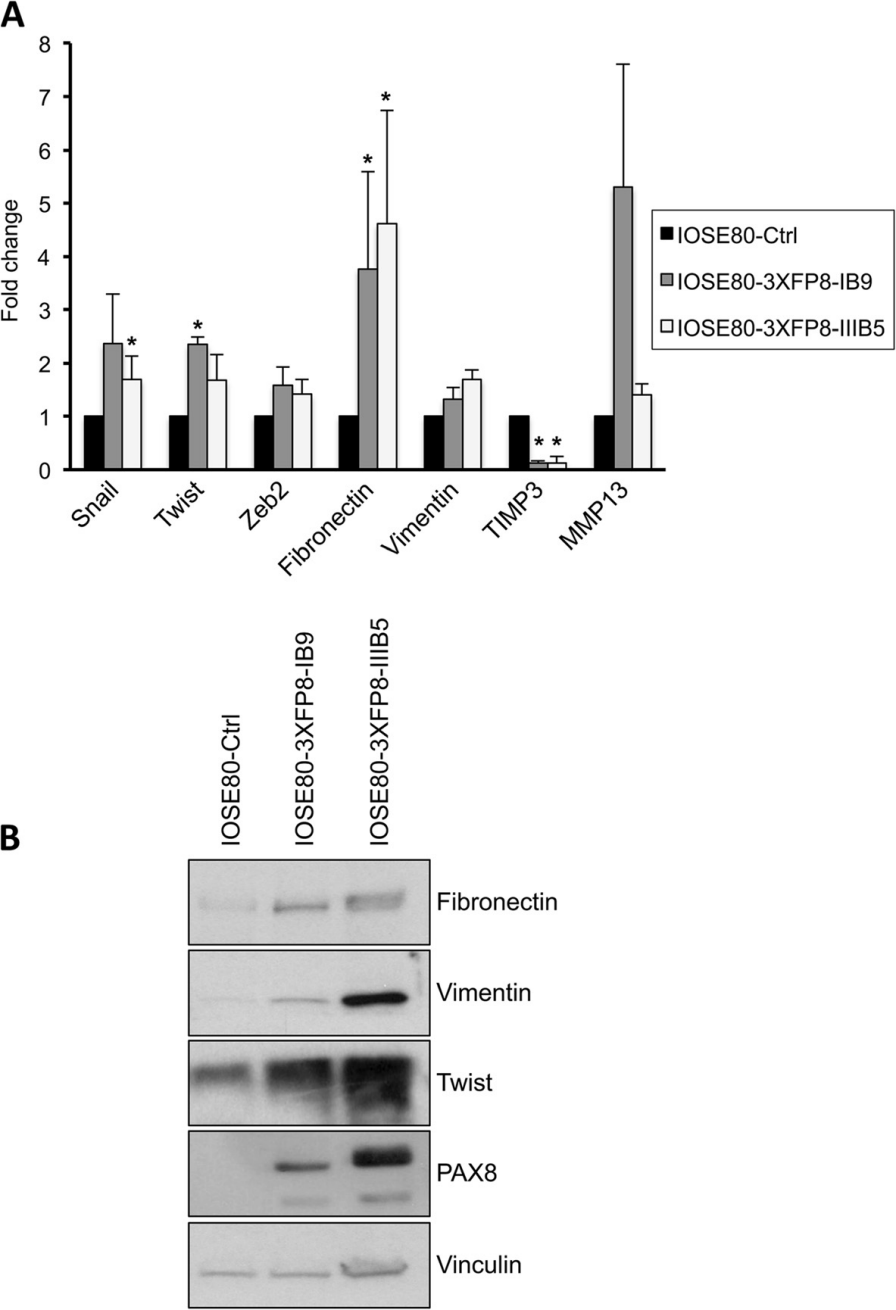
**Expression of PAX8 induces EMT in normal ovarian cells. (A)** Quantitative PCR for Snail, Zeb2, Twist, Fibronectin, Vimentin, MMP13 and TIMP3 in IOSE80-3XFP8-IB9 and IOSE80-3XFP8-IIB5 clones (IOSE-80PC cells transfected with 3XFLAG-Pax8) and in IOSE80-Ctrl (IOSE-80PC transfected with the backbone vector). *Columns,* mean of three independent experiments in duplicate; *bars,* SD. *, Ρ < 0.01. **(B)** Total protein extracts of IOSE80-Ctrl cells and independent stable clones IOSE80-3XFP8-IB9 and IOSE80-3XFP8-IIB5 were separated on SDS-PAGE and subjected to Western blot analysis with specific antibodies as indicated in the panel. The hybridization with vinculin assessed the protein uniform loading and integrity.

Having previously established that PAX8 enhances motility, invasion and tumor formation, we hypothesized that it could also play a role in epithelial-mesenchymal transition (EMT). To test this hypothesis, we stably transfected the IOSE-80PC normal ovarian cell line with full-length PAX8 and isolated several independent clones. By Q-PCR and Western blot, we analyzed the expression levels of epithelial and mesenchymal markers in two representative clones (IOSE80-3XFP8-IB9 and IOSE80-3XFP8-IIIB5). Although E-cadherin expression could not be detected in these cells, several genes that act as E-cadherin repressors, such as Snail, Twist and Zeb2 [19] were significantly upregulated in PAX8 overexpressing IOSE-80PC stable clones compared to control mock transfected cells (Figure 4A and B). At the same time, the expression levels of some mesenchymal markers like fibronectin and vimentin were significantly increased (Figure 4A and B). In addition, we measured the expression levels of TIMP3, which were strongly decreased in PAX8 overexpressing IOSE-80PC cells (Figure 4A), in agreement with recent data indicating that TIMP3 is negatively regulated by SNAIL [20]. The metalloproteinases MMP2 and MMP9 are also regulated by SNAIL [20]; however, in our cells, no significant changes in the expression of these metalloproteinases were observed (data not shown). At the same time, the expression level of MMP13 was significantly upregulated suggesting that in different contexts, other metalloproteinases could be regulated by SNAIL.

## Discussion and Conclusions

Epithelial Ovarian Cancer (EOC) is a morphologically and biologically heterogeneous disease and remains a leading cause of morbidity and mortality. It accounts for approximately 3% of all cancers in women and despite considerable efforts to improve early detection and advances in chemotherapy, the highest mortality rate of ovarian cancers has markedly increased worldwide.

There is ample evidence that dysregulated expression and/or activation of specific members of the PAX family appear to play a major role in the progression of specific cancers arising in those organ systems in which PAX proteins exert their developmental functions during embryogenesis [21], but their precise role in cancer is still unclear. Recently, a genome-scale analysis of 102 cancer cell lines identified PAX8 as a lineage-specific survival gene, highly expressed in ovarian cancer lines and amplified in a substantial fraction of primary ovarian tumors [22]. It was initially hypothesized that epithelial ovarian cancer derives from the epithelial cells covering the ovary, but recent evidences showed that high-grade serous carcinoma (HGSC) originates in the fallopian tubal secretory epithelial cells, which are positive for PAX8 expression [15]. Newly, we have demonstrated that PAX8 plays a critical role in cell cycle progression and cell survival of differentiated epithelial cells [23], reinforcing the crucial involvement of this transcription factor in different biological processes. To investigate the role of PAX8 in ovarian cancer we performed studies *in vitro* and in *vivo*. We analyzed the expression level of PAX8 in a series of ovarian cancer cell lines, thus we have chosen SKOV-3 cell lines to assess PAX8 involvement in ovarian tumorigenesis. We selected stable cell clones constitutively silenced by sh-PAX8 to detect the function of this transcription factor in an epithelial ovarian cancer cell line. Our results indicated that PAX8 knock-down elicited a dramatic effect on SKOV-3 cell growth, inhibited the invasion rate of these cells through the Matrigel, and reduced the migration rate in wound-healing assay. To assess the ability of PAX8 to inhibit tumor growth *in vivo,* we injected SKOV-3 cells constitutively silenced by shPAX8 into nude mice. The results obtained in this study show for the first time that PAX8 is capable of inducing *in vivo* tumor growth. The size of palpable lesions well correlates with PAX8 expression level of single clones, confirming the role of PAX8 as oncogene *in vivo.*

Recently, consistent with our findings, it was reported that PAX8 transcriptionally regulates E2F1, a key regulator of the G1/S phase of the cell cycle [24]. To date, the essential role of PAX8 in the development and differentiation of the thyroid gland has been extensively described; nonetheless, its role in other contexts has not been addressed. Our data provide the first evidence of a clear involvement of PAX8 in the *in vivo* tumorigenesis of ovarian cancer cells. Given the enormous heterogeneity of ovarian cancer and the enhanced expression of PAX8 only in some epithelial subtypes, it will be very interesting to see if a subset of novel PAX8 target genes is relevant for cancer initiation and/or maintenance, in order to identify novel targets for ovarian cancer therapy.

To further characterize PAX8 effects on cell migration and invasion we analyzed the expression level of some master regulators of EMT [19] in normal ovarian cell line IOSE-80PC stable transfected with PAX8. Here, we reported that the expression of PAX8 significantly induces SNAIL and MMP13 while inhibits TIMP3, reinforcing the idea that this transcription factor might represent a potential new target for preventing ovarian tumor invasion and metastasis. In conclusion, we believe that the role of PAX8 in cancer is emerging as an exciting research area and promises to deliver many new insights into the onset and growth of ovarian epithelial carcinomas.

## Competing interests

The authors declare no conflict of interest.

## Authors’ contributions

DPT and ZM designed research; DPT, LV, dCT and FMG performed research; DPT and ZM analyzed data, DPT and ZM wrote the paper. All authors read and approved the final manuscript.

## Pre-publication history

The pre-publication history for this paper can be accessed here:

http://www.biomedcentral.com/1471-2407/14/292/prepub
